# New Jersey State Dental Society

**Published:** 1884-10

**Authors:** 


### NEW JERSEY STATE DENTAL SOCIETY.

The fourteenth annual meeting of this Society was held at Asbury Park, on July 16th, 17th and 18th. There was a large attendance and a very interesting meeting. Seven new members were admitted to the Society, which now numbers 98 active members.

From the report of the State prosecutor and the discussion thereon, appears the significant fact that there has never been a conviction under the law, notwithstanding the best possible evidence, it would seem, has been produced against its violators. In a case tried during the last year, where the victim of the unlicensed practitioner's mal-practice and the tooth he filled were produced in court, the jury failed to convict, and subsequently the prosecutor was sued for malicious prosecution. The efforts to enforce the law have, nevertheless, generally resulted in the offenders leaving the State, quitting practice, or becoming qualified as the law requires.

Drs. Geo. C. and G. Carleton Brown presented several models and diagrams of cases of regulating teeth by means of Dr. Patrick's regulator, which they described at length.

*Dr. G. Carleton Brown*—Said, the first case is that of a young lady of about twenty-two years. The mouth was so very narrow through the centre that the front teeth projected far out of the mouth, so that the upper lip would not cover them. Our first plan was to spread the arch, and for that we used a modification of the Coffin plate, making a black rubber plate to fit accurately over the teeth, bearing a little harder on the bicuspids and molars, with a piano wire spring in the centre. This was put on about July 30th and continued until October 26th. We then applied the Patrick apparatus, and drew the two bicuspids out and the incisors in. The principle of the thing is a gold spring or bar, made as near as possible the shape of the arch, which is attached to the molars and passes around outside, and the idea is to bring the teeth to this line. The case was discharged April 1st, the teeth being in perfect line. This second case was commenced January 14th, 1884. The first bicuspid was extracted in order to gain room. The case was discharged April 3d, the teeth being in good line except the canine, which has not come down far enough to be drawn out. It is claimed by Dr. Patrick that he can regulate any case of any kind with his apparatus. It may be that he can, but I doubt it. I do not think it possible, with one appliance, to regulate all cases, but it is as near a universal regulator as I have yet seen.

*Dr. Geo. C. Brown*—This case, No. 3, gave us more trouble than any other. The right lateral was very much out of position, but by tying a ligature of floss silk around the neck and passing it around the bar, the tooth was readily turned. The left lateral was of very peculiar shape, and it was impossible to get a ligature attached to it. We had great difficulty in fastening the gold band around the tooth, owing to its peculiar shape. Finally, by putting soft oxy-phosphate over the tooth and pressing the band over that, and holding it until the oxy-phosphate hardened, the band was secured as nicely as could be desired. By putting the band on with oxy-phosphate in this way and attaching the rubber to the arm of the band, you can turn a tooth clear around, or to any position you please, and there is no danger of the band becoming loose. You can apply the Patrick regulators to almost any kind of case, and a little ingenuity in adapting it to the case will enable you to bring the teeth into the required position. The Drs. Brown described other similar cases.

*Dr. S. G. Wallace*—Described a case of regulating loose teeth. The inferior centrals and laterals were very much loosened by an accumulation of tartar. He removed the tartar, extracted the left lateral, which was too loose to be saved, and proceeded to bring the others into line and close up the space by the positive method. For closing up the space he used French tubing over the central and lateral, letting it come down to the margin of the gum and securing it with ligatures. He then made a gold band for the outside, similar to Patrick's, attaching it to the first bicuspid on each side, also a similar band for the inside of the circle, attaching it to the canines. The centrals and lateral stood edgewise, the approximal surfaces inclined inward. Before adjusting the bands a piece of tubing was slipped over both, so that when in position the tubing was drawn through the space where the tooth was extracted. This held the bands in position, and the teeth were drawn into line and the space entirely closed up in ten weeks' time. When he commenced the case the gum and process were much absorbed and the four incisors so loose that there was doubt about saving any of them. It is two years since the case was completed; he saw it yesterday, and found the mouth in a healthy condition, the tissues somewhat restored, and the teeth and gums perfectly solid. He treated the gums during the operation, and saw the patient five times during the ten weeks. He has pursued this method in many cases with very successful and satisfactory results.

*Dr. C. S. W. Baldwin*—Four years ago, before I had heard of the Patrick regulating system, I applied an apparatus, made very much on the same principle. I made a silver band and attached to it at either end pieces of rubber that fitted the molar teeth perfectly; two holes were drilled through the rubber, and it was tied to the molars. The teeth I had to regulate were the central, lateral and cuspid. The lateral stood much inside of the arch, the central had to be twisted, and the cuspid had to be drawn in. The natural tension of the band drew in the cuspid very readily. I used rubber tubing to draw the lateral out; and to twist the central I used a cord formed into a slip-noose and so attached as to draw on the proper side of the tooth, the other end being tied to the band. I think I was something like seven months in regulating that case. I had great difficulty in getting the patient to wear this band on the outside of the teeth; she was a young lady, and naturally objected to the disfigurement. It was a very successful and satisfactory case of regulating.

*Dr. F. C. Barlow*—I have a case somewhat similar to that which I have been working on for some time with metal plates, and without much success. I found it very difficult to turn the teeth. Last week I took hold of Dr. Patrick's apparatus, and I have it on the case now. The patient does not object to wearing it.

*Dr. B. F. Luckey*—I have been using the Patrick regulator for the last three or four months in drawing out teeth, and also in rotating them, and with excellent success.

*Dr. C. S. Stockton*—I have never used the Patrick regulators; nevertheless I am very much interested in the remarks of the Drs. Brown, for the reason that this apparatus is one that you can buy at the dental depots and have ready at your hand, and is therefore a great convenience. I have used for drawing in projecting front teeth an apparatus something like it, which patients can wear and adjust with more ease and comfort than they can this. If you take out the first bicuspids, as I did some time ago in a case, apply a band around the first molars with a nut attachment, then extend a band around the arch with a screw attachment; you can give your patient a little key, with directions to give that screw a turn once a day until the pressure is felt. The case I speak of was regulated in about six weeks, the space being entirely closed up. I saw the patient but once a week, and then did nothing; the patient does the whole work. I have two cases of that kind now. In one I took off the apparatus in about four months, and one is wearing it now. Dr. Dodge, of New York, recently reports a case of a gentleman who had worn away the back teeth so that the front teeth had been forced out of position, and when the back teeth had been built up and restored so that they articulated as they formerly did, these teeth fell back after the lapse of twenty years to what was their normal position. Therefore the question arises: How long must you wear these regulators in order to bring teeth into position and *keep* them there? Both of these cases I have spoken of have been very satisfactory to the patient. Nothing is more disfiguring to a person than protruding front teeth, and there is no work the dentist has to do which gives more pleasure to his patients or makes them firmer friends than the regulating of such cases. Take the case spoken of by Dr. Brown, where he restored the beauty of his disfigured patient so that she could be mistaken for her always pretty sister; nothing he could do for that woman would secure her gratitude so surely as that. I have had more pleasure and profit in using the Coffin plate than in using any other regulating apparatus. I saw Dr. Coffin in London, and he took the trouble to show me a large number of models, over one hundred certainly, of cases of regulating, and from an examination of them I am convinced that the cases are very few where you cannot use his device. Dr. Watkins had a case some time ago, which I saw in his office in Montclair, where he wanted to draw in the lower front teeth, and he made a piece of that kind. He covered the molars and bicuspids with a rubber plate, which is a very necessary part of the operation, having a bar running across inside, and the teeth were drawn in by means of rubber bands attached to them and to this bar. I regulated a case in a similar manner some time ago, using piano wire for the bar. By covering the teeth that are not to be moved they are held firmly in position; there is no giving way; the bands and wires pull and push the mal-positioned teeth where you want them, while the rubber plate holds the others in position securely, making a basis from which to draw the wanderers into place. Without having had experience in the use of Dr. Patrick's apparatus, I know of no other that is equal to Dr. Coffin's.

*Dr. S. C. G. Watkins*—As Dr. Stockton has mentioned my name I will tell you about the case he referred to. It was a boy about twelve years of age, whose lower incisors closed outside of his upper ones so that the upper incisors touched the lower gum inside. His chin was thrown away forward, and his face had a very peculiar expression, as you can readily imagine. I applied a plate which covered the lower back teeth so as to give a grinding surface and open the mouth sufficiently to allow the lower incisors to pass by the upper ones. I placed rubber rings on a bar that runs across the plate inside, and tied silk floss around the projecting teeth, and secured that to the rubber rings so as to draw them tight. By using silk floss instead of rubber around the teeth you are not bothered by the band working down under the gum and causing inflammation, because the floss will stay just where you put it. In this case I saw the boy once a week for nine weeks, changing the apparatus and cleaning each time. At the end of nine weeks I removed the apparatus, telling the boy to close his mouth and keep it closed, so that the upper incisors would close over and prevent the lower ones from returning to their former position, if there should be any such tendency. The effort of the lower teeth to get back to their old position pressed the upper ones out considerably. The lower ones moved back so that they stood erect. I took the apparatus off last January, I think, and everything is lovely so far. They cannot get back unless they crowd the upper teeth far out, which is not likely to happen. The boy's speech is very much improved.

*Dr. W. H. Atkinson*—I am delighted to see the progress that has been made, and the intelligent expression of the knowledge of principles that has been manifested here this afternoon. One important point in regulating teeth has been overlooked. Wherever teeth in their occlusion hold other teeth out of line, the mouth must be opened far enough to relieve that point. When that is done teeth can be transferred to any extent, and without even tenderness, much less pain. Wherever you have tender teeth in these operations, you have done your work imperfectly and unintelligently. The point is well taken as to the utility of the bars that are used externally, and which describe the arc of an ellipse in which the teeth should stand, and every method which looks in that direction has more or less intuitive intelligence in it ; but the great point is to find the teeth that are not to be moved, if any do occlude rightly, and if they do not, they should be brought into position. When that has been done they should be held there until reconsolidation of the alveolar process takes place. What is called decalcification, or solution of the lime-salts, has to take place every time you remove a tooth ; and if you hold the teeth in position until reconsolidation takes place, then when irritation comes on you do not get such an example as that referred to by Dr. Stockton, of teeth wandering after twenty years, if you have your occlusion right, so that each of them may do its share of the work. Dentists are too much afraid of using the corrundum wheel in trimming the points of teeth that have not been worn down so as to be equal with those that have been worn. They do not see that the teeth occlude in a proper manner so as to perform the function of teeth. In moving teeth we need not take nine months to do it, nor nine weeks, if you follow the line of instructions. Take your impression and make a model of the mouth, and then study your case. Make up your mind what teeth are not to be moved, then make a plate that will give you an occlusion to chew upon, and to this attach a bar of platinum or iridium, passing around the outside of the arch ; mark on this bar the points to which each tooth is to be drawn, and there punch holes through the bar for the reception of the rubber bands that are to draw the teeth in the required direction, and never change it. It is better to make the holes into slots by sawing out the upper part of the bar down to the holes, to prevent the checking of the rubber rings; countersink each side of the holes, and with a corrundum bur polish them off nicely, the idea being to keep the band that goes around the neck of the tooth from going far enough to interfere with the dental ligament or festoon of the gum, but just far enough to get hold of the tooth. Slip the other end into the little slots in the bar. The teeth are to be drawn out to the required position plus a little, so that there will be a little room for them to fall back; and when they fall back they will all be occluded, each one against its antagonist, and stand like staves in a barrel; and being like staves, chamfered a little, they will probably not need anything in the way of coopering at all; the occlusion will hold them in place. If you trim the teeth that are not worn down to conform to those that are, the occlusion will be perfect. Then apply a little tincture of calendula and dismiss your patient for a week. It is not the amount of pressure that you bring to bear upon a tooth that accomplishes the object you have in view, but the continuousness of it. My soul yearns when I think of twisting and turning away for three or four or five years to get a tooth into position, as we used to do. Let the machine do its work, and there will be no tenderness of the teeth if you do not keep biting upon them. What men call inflammation is not inflammation at all; it is simply a retrograde metamorphosis of the tissues so as to make them soft enough and near enough to their embryonic condition to be guided into the position required by the new circumstances, and the process of reconsolidation of the softened sockets is a repetition of the metamorphosis from the embryonic condition to the adult state of the tissues. If there is any disposition of the moved teeth afterwards to return to their former position, you may be sure you have not understood your case. Cases in which extraction is desirable are so rare that they do not deserve attention. In just a few cases of heredity, where there has been a crossing of diverse races, it may be necessary, but, as a rule, in these cases of irregularity there is nothing to warrant the extracting of teeth.

*Dr. M. W. Foster*—Did I understand you rightly that you apply direct ligatures to the teeth?

*Dr. Atkinson*—Yes, sir.

*Dr. Foster*—And without tenderness?

*Dr. Atkinson*—And without tenderness.

*Dr. Trueman*—Will you explain how you keep the rubber from impinging upon the soft parts?

*Dr. Atkinson*—I will. By having the bar stand in such a position as to prevent the rubber from creeping against the festoon of the gum.

*Dr. Geo. C. Brown*—I understood you to say that teeth can be moved in less than nine weeks, and without causing inflammation?

*Dr. Atkinson*—I did.

*Dr. Brown*—How is it that we have so much trouble in separating teeth with rubber for the purpose of filling, and how would you suggest that the operation be performed so as not to produce soreness?

*Dr. Atkinson*—Without any pushing.

*Dr. Brown*—I don't care how little pushing you do, when you move teeth they will be sore.

*Dr. Atkinson*—You are very much mistaken. I have seen teeth separated with rubber so that I could put my little finger in the space. If you but understand the histological and pathological conditions of the tissues you will know what makes this tenderness, and you need not have it at all, even in children.

*Dr. Brown*—Can you separate teeth sufficiently wide for filling without tenderness?

*Dr. Atkinson*—Yes.

*Dr. Brown*—In how long a time?

*Dr. Atkinson*—In five minutes.

*Dr. Brown*—What with?

*Dr. Atkinson*—With wedges. The man who will separate teeth day after day for the purpose of filling is to be pitied.

*Dr. Brown*—I pity the patient of the man who does it in five minutes.

*Dr. C. S. Stockton*—In the case I spoke of where the upper incisors protruded over the lower ones so much that you could place your finger between them, it was necessary, in order to make a successful operation, to extract the first bicuspids. Dr. Atkinson's idea is that only in very rare cases is it necessary to extract teeth, which is true, but this was one of those cases where a man who says his prayers does extract teeth; a case, may-be, where a Dutch woman had married an Irishman, or something of that kind.

*Dr. Atkinson*—If Dr. Stockton says it is so I am quite willing to believe it, and consider it one of the exceptions to the rule.

*Dr. James Trueman*—Without intending offence to Dr. Atkinson, I wish to enter my protest against the doctrine he has enunciated. That teeth can be moved and the periosteum be irritated sufficiently to produce what we call absorption without producing soreness of the teeth is an absurdity that I never before heard promulgated in a dental convention. I contend that it is impossible to move teeth without irritating the periosteum, and that irritation will necessarily produce more or less soreness. In regard to wedging teeth, which he advocates so strongly, to my mind wedging teeth, and doing it in five minutes, is one of the worst features ever introduced in our profession. You cannot do that without producing irritation. You can move them a short distance by reason of the elasticity of the periosteum, but when you move them more than that you produce destruction, and you get strangulation of the pulp. You can move teeth slowly and produce very little irritation, but there is always irritation, and always inflammation as far as it goes.

*Dr. Atkinson*—It takes intelligence to comprehend. How does the gentleman get the elasticity of the periosteum that he speaks of? It is not the elasticity of the fibrous connective tissue of the periosteum, but the pressing of the neurine in the fine nerve filaments and the blood in the capillaries that are distributed through the meshes of this connective tissue that give way by pressure, and thus set up a retrogressive metamorphosis, by which the lime-salts in the sockets are dissolved and the teeth allowed to move. I did not say I would move teeth at one clip, but that I would do it in five minutes. Take the stiffest teeth, put in a wedge gently at the cutting edge so as to strain just a little, then insert one near the gum and tap it gently until the first one has been relieved, and so by successive taps upon each wedge alternately you will get the room you want. How do you get that room? You get it by mechanically impinging upon the vessels that carry the fluids through the fibrous connective tissue of the periosteum, and you get it without inflammation and without irritation. Men say "inflammation" and "irritation" to cover up the d——d ignorance that cannot tell what inflammation or irritation is. Irritation means scratching, and sometimes means a rebuke. When you move a tooth slowly, the blood that is in the capillaries and the neurine that is in the nerve filaments are expelled from every one of the nerves and capillaries, and when the pressure is relieved, those currents will come back without the faintest blush of tenderness.

(TO BE CONTINUED.)

